# Evaluation of inspiratory pressure in children with enlarged tonsils and adenoids

**DOI:** 10.1016/S1808-8694(15)31263-5

**Published:** 2015-10-20

**Authors:** Melissa Guerato Pires, Renata Cantisani Di Francesco, Anete Sevciovic Grumach, João Ferreira de Mello

**Affiliations:** 1 Specialization in Respiratory Physical Therapy, Physical Therapist.; 2 Ph.D. in Medicine, Medical School, USP, Assistant Physician, Ph.D., Hospital das Clínicas, Medical School, USP.; 3 Ph.D. in Pediatrics, Physician, Ambulatory of Primary Immunodeficiency; Researcher, Laboratory of Medical Investigation on Allergy and Immunology, Department of Dermatology, FMUSP.; 4 Ph.D. in Medicine, Medical School, USP. Collaborating Professor, Discipline of Otorhinolaryngology, Medical School, USP.

**Keywords:** tonsil, adenoids, child, adenoidectomy, tonsillectomy

## Abstract

Children with enlarged tonsils and adenoids usually present breathing abnormalities such as snoring, mouth breathing and sleep apnea. It is known that upper airway obstruction and consequent mouth breathing may result in pulmonary diseases. **Aim**: The goal of this preliminary study was to evaluate the inspiratory pressure in children with upper airway obstruction due to enlarged tonsils. **Study design:** clinical with transversal cohort. **Material and Method**: We evaluated 37 children (4 -13 years old, female/male) with enlarged tonsils who would be submitted to a T&A surgery in the Department of Otolaryngology, Medical School, University of Sao Paulo, from October 2002 to March 2003. The control group comprised 28 children without tonsillar disease submitted to the same tests. Inspiratory pressure was obtained using a manometer and vacuum meter. **Results**: We could observe lower inspiratory pressures in children with upper airway obstruction. The mean of inspiratory pressure in the upper airway obstruction group was 14.607cm/H2O and in the control group was of 27.580cm/H2O. **Conclusions**: Enlarged tonsils and adenoids were associated with poor inspiratory pressure, resulting in increased breathing effort and work of the involved muscles.

## INTRODUCTION

Upper airway obstruction due to enlarged tonsils results in limited airflow. Such limitation is caused by a mechanical blockage that obstructs airflow, leading to mouth breathing, as it has lower resistance to air passage. ^1^

Under normal conditions, nasal resistance is greater in childhood – nearly six times higher than that observed in adults. According to Barreto^2^, nasal airway accounts for 2/3 of total respiratory resistance.

Pulmonary repercussion due to tonsil enlargement is better understood if considered in the context of “united airways”. In fact, disorders of upper and lower airways frequently coexist, as they present similar histology 3, 4.

Several clinical complaints have been associated with upper airways obstruction, such as excessive day sleepiness, desynchronized sleep, day headaches, aggressiveness, restless sleep, deep sudoresis, night enuresis, weight-height deficit^15^. Some authors relate obstruction of upper airways to bruxism. ^6^

Cazerta and Pacheco^7^ described two children with *cor pulmonale*, pulmonary edema and respiratory difficulty due to obstruction of airways by enlarged tonsils. After surgery, disorders regressed in one child, while the other child evolved to death due to important and irreversible hypertrophy of the right ventricle. Andrade& Britto reported five children with cardiac disorders due to enlarged tonsils, who presented regression of disorders in all cases^8^.

Studies designed to objectively assess pulmonary repercussion of enlarged tonsils have not been developed. Several evaluation methods have been proposed to quantify the strength of respiratory muscles^9^.

Inspiratory Pressure (IP), measured by a manometer and vacuum meter, is the most common and easy parameter. The manometer plus vacuum meter is used to quantify positive pressures (manometer) and negative pressures (vacuum meter).

Measurement of respiratory muscles strength may be widely employed, allowing diagnosis of respiratory insufficiency due to muscular failure, early diagnosis of respiratory muscle weakness, helping the evaluation of respiratory mechanics and indication of intubation, removal of artificial breather and patients’ extubation.

Due to the importance of efficient breathing in children with enlarged tonsils and the lack of objective data on this disorder’s repercussions, the present study aims at assessing IP in children with enlarged tonsils.

## MATERIAL AND METHOD

Thirty-seven children (age range of 4-13 years, female/male) with obstruction of upper airways by enlarged tonsils were evaluated and followed up in the Department of Otolaryngology, Medical School, University of Sao Paulo, from October 2002 to March 2003.

Two children were excluded – one female and one male – for not performing in the procedure, and 7 children for presenting grade 2 enlarged tonsils. Ten children were excluded, as they were under 6 years of age, since kids in this age were not evaluated in the control group.

Children evaluated in the control group came from *Casa de Apoio Madre Clélia*, were within the same age range of the studied group and did not present tonsil enlargement or other respiratory disorders. IP tests were performed similarly and following the same criteria as those used in the enlarged tonsils group.

In the control group, 28 children were evaluated (11 females and 17 males) in the 4-13 age range. Three (3) children were excluded – 2 did not understand what was being asked and 1 presented tonsillitis at evaluation. Diagnosis of enlarged tonsil was obtained through paranasal sinuses radiography. Severity of palatine tonsillar obstruction was classified according to criterion described by Brodsky^10^ (Table 1), including patients with grades III and IV obstruction.

**Table 1:** Grade of obstruction of palatine tonsil according to Brodsky^10^.

Grade	Proportion of Tonsil in Oropharynx
0	Tonsil in Palatine Fossa
1	Tonsil occupying less than 25% of oropharynx
2	Tonsil occupying 25 - 50% of oropharynx
3	Tonsil occupying 50 - 75% of oropharynx
4	Tonsil occupying more than 75% of oropharynx

All the assessed children were in the group with surgery indication (adenotonsillectomy). Patients excluded from the group were those with asthma, neurological involvement, no surgery indication or those who did not understand what was being asked or did not help during complementary exams.

Inspiratory pressure (IP) is defined as a maximum negative pressure produced orally against occluded airway^11^, as well as the maximum negative pressure through the mouth after complete expiration of residual volume, followed by a single inspiration of maximum effort^12^ or diaphragm force index^13^. This measure was obtained by a MV-120 manometer plus vacuum meter (*Ger-Ar-SP Com. Equip. Ltda*.) by means of a tracheal and mouth connector. For final results, all children had 3 attempts and the highest value was considered (cm/H2O). IP value was obtained with the children comfortably seated, with no restrictions to pulmonary expansion, such as tight clothes and orthodontic braces, among others.

The protocol was submitted and approved by the Ethics and Research Committee of the Otorhinolaryngology Department, *Hospital das Clinicas*. Parents’ permission term was signed before evaluation.

## RESULTS

**Graph 1:**
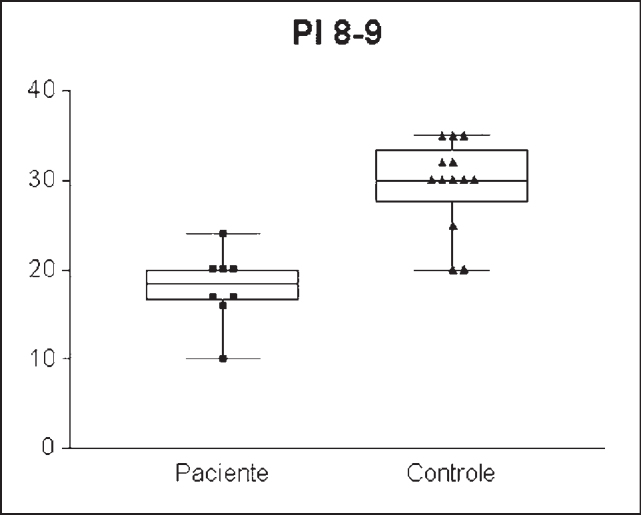
Inspiratory pressure of children between 8 and 9 years old. IP = Inspiratory pressure. 8-9= age between 8 and 9 years.

**Graph 2:**
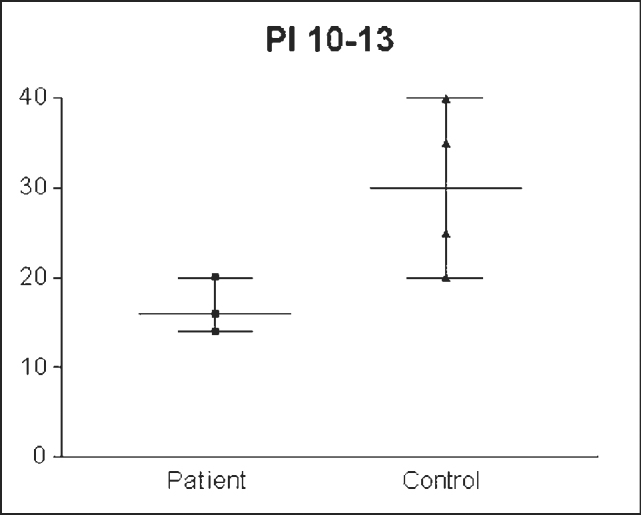
Inspiratory pressure of children between 10 and 13 years old. IP = Inspiratory pressure. 10-13= age between 10 and 13 years.

**Graph 3:**
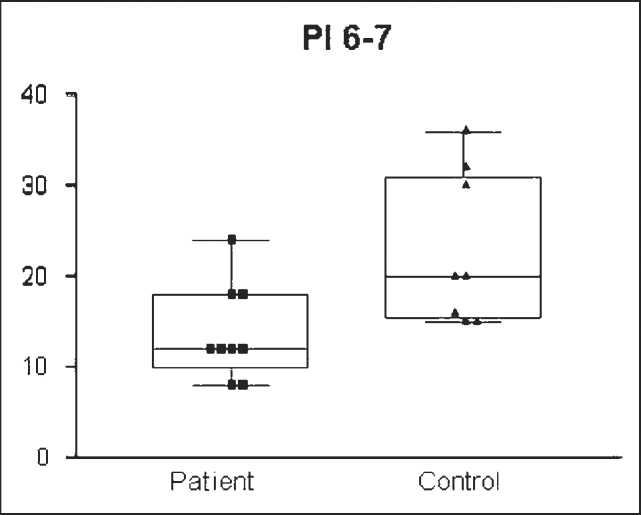
Inspiratory pressure of children between 6 and 7 years old. IP= Inspiratory pressure. 6-7= age between 6 and 7 years.

**Graph 4:**
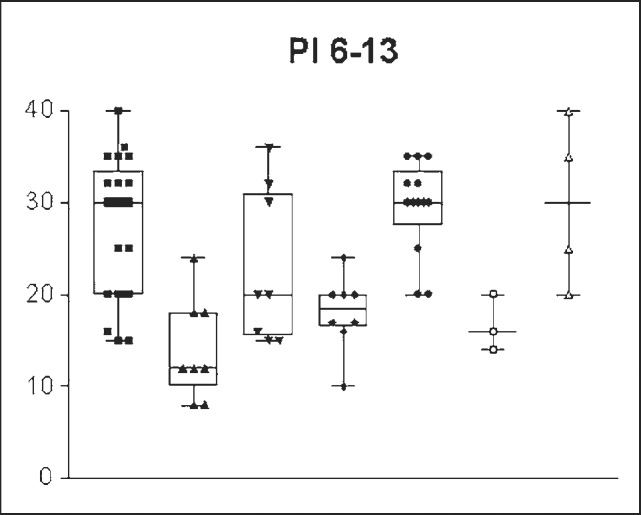
Inspiratory pressure of children between 6 and 13 years old. IP= Inspiratory pressure. 6-13 = age between 6 and 13 years.

The IP value obtained was significantly different between the groups: 14.607cm /H2O ± 7.3321 (8-24 cm/H2O) in the enlarged tonsils group and 27.580/H2O ± 4.7791(15- 40.00 cm/H2O) in the control group (n= 0.001) (Graph 1). Standard error was of 1.4664 in the enlarged tonsils group and of 0.8880 in the control group. Groups’ assessment according to age ranges presented the following: PI = 13.777cm/H2O ± 5.2387 (8-24cm/H2O) in the enlarged tonsils group (n=8) and 23.0 cm/H2O ± 8.4007 (15-36cm H2O) in the control group (n =7) among children between 6 and 7 years of age, with statistically significant difference (p= 0.003) (Graph 2). Inspiratory pressure analyzed in the 8-9 age range presented an IP of 18.00cm/H2O ± 4.106 (10 – 24.00 cm/H2O) in the enlarged tonsils group (n=8) and IP of 29.54cm/H2O ± 5.043 in the control group (n=13) (p= 0.001) (Graph 3). The enlarged tonsils group in the age range of 10-13 years presented IP of 16.67cm/H2O ± 3.055 (14 – 20.00cm /h2O) (n=3). Statistical difference was not significant between both groups (p= 0.293) (Graph 4).

## DISCUSSION

Tonsils enlargement is one of the main causes of respiratory disorders during sleep. Oral breathing and snoring are very common symptoms in children^14^.

There are no reports describing the use of IP for evaluation of respiratory muscle strength in children with enlarged tonsils. Therefore, we searched for groups of study on different pathologies in which IP measurement was applied in other diseases as a parameter of secondary pulmonary involvement.

Iandelli assessed patients with neuromuscular diseases and verified several factors that may influence the IP value, such as: different techniques, personal motivation, cooperation and facial muscular weakness. However, despite these variations, this parameter may be applied in the evolution process of disease, as well as in the response to therapy. ^15^

In myasthenia gravis, the evaluation of 23 patients demonstrated that respiratory muscle strength remained normal, with no restrictive standard; however, the strength of respiratory muscles was reduced. ^16^ In patients with multiple sclerosis, inspiration pressure is jeopardized by fatigue, muscular spasticity, and lack of coordination of respiratory and facial musculature^12^. In normal subjects, where the efficacy of postoperative incentive inspirometer was assessed, IP value was a good way to measure gain of the respiratory activity and, consequently, respiratory muscle strength^17^.

In the present study, children with enlarged tonsils presented lower IP than those of the control group, which suggests reduced respiratory activity and, consequently, lower oxygenation, resulting in chronic hypoxia, as reported by Cazerta. ^7^

Children with enlarged tonsils presented difficulty to mechanically breathe through the upper airways and, to survive, they chose a less resistant air passage, that is, the mouth. However, with time, they make less effort to breathe, demanding less force from respiratory muscles, which leads to muscle weakness. This mechanism explains the different pressures among the groups.

The IP difference is more evident in younger age ranges. It is known that 90% of the craniofacial growth occurs up to 12 years old^5^. Therefore, at this age, when tonsil enlargement occurs, adjustment of bone structures regarding palatine and/or pharyngeal tonsils have already occurred. Children with enlarged tonsils present lower inspiratory pressure, which in our study was more evident among those at ages of 6 and 7 years.

The children included in our study, as well as the patients evaluated by Diez in a study conducted at Hospital La Paz – Madrid, in which 23 patients with clinically stable Myasthenia Gravis were assessed^16^ , did not present respiratory fatigue, despite the weak musculature. In both groups, IP and EP values were analyzed.

We concluded that children with enlarged pharyngeal and palatine tonsils presented lower inspiratory pressure. These children must be treated, once tonsillar enlargement, in the long term, leads to facial tonus alterations, morphological craniofacial and occlusive alterations, with reduction of respiratory muscle strength. Moreover, the quality of life of these children is jeopardized.
